# Identifying the Threshold of Dominant Controls on Fire Spread in a Boreal Forest Landscape of Northeast China

**DOI:** 10.1371/journal.pone.0055618

**Published:** 2013-01-31

**Authors:** Zhihua Liu, Jian Yang, Hong S. He

**Affiliations:** 1 State Key Laboratory of Forest and Soil Ecology, Institute of Applied Ecology, Chinese Academy of Sciences, Shenyang, China; 2 School of Natural Resources, University of Missouri, Columbia, Missouri, United States of America; DOE Pacific Northwest National Laboratory, United States of America

## Abstract

The relative importance of fuel, topography, and weather on fire spread varies at different spatial scales, but how the relative importance of these controls respond to changing spatial scales is poorly understood. We designed a “moving window” resampling technique that allowed us to quantify the relative importance of controls on fire spread at continuous spatial scales using boosted regression trees methods. This quantification allowed us to identify the threshold value for fire size at which the dominant control switches from fuel at small sizes to weather at large sizes. Topography had a fluctuating effect on fire spread across the spatial scales, explaining 20–30% of relative importance. With increasing fire size, the dominant control switched from bottom-up controls (fuel and topography) to top-down controls (weather). Our analysis suggested that there is a threshold for fire size, above which fires are driven primarily by weather and more likely lead to larger fire size. We suggest that this threshold, which may be ecosystem-specific, can be identified using our “moving window” resampling technique. Although the threshold derived from this analytical method may rely heavily on the sampling technique, our study introduced an easily implemented approach to identify scale thresholds in wildfire regimes.

## Introduction

Fire is an integral component of many terrestrial ecosystems [1], one that shapes vegetation structure [2], [3] and plant traits [4]. In boreal forests, fire is mainly influenced by fuel, weather, and topography, but the relative importance of these influences is controversial [5]–[9]. Some studies have noted that fire is mainly dominated by weather, especially during severe weather events that drive large fires (hereafter: weather hypothesis) [5], [9]. Supporters for the weather hypothesis argue that fire driven mainly by weather consumes different fuel types indiscriminately; therefore, the influence of fuel on fire is negligible. By contrast, some studies demonstrated that fuel also influences fire, even under extreme weather conditions [6], [8], by filtering and modifying fire behavior (hereafter: fuel and weather hypothesis) [10]. Advocates for the fuel and weather hypothesis believe that fire will burn preferentially in some fuel type over others; therefore, vegetation and fuel types less susceptible to fire can be used as fire breaks to slow the rate of spread or lessen the intensity of fires, and to aid suppression efficiency.

Fire spread is a spatially contiguous process driven by controls acting across a range of scales [11], [12]. Scale refers to the spatial extent of an ecological process, such as the extent of fire spread (fire size) considered in this study. At a fine scale, local distribution of flammable fuels and topography (bottom-up controls) determine when and where a fire occurs, and subsequently its rate and direction of spread. As fires grow in size, they reach points where future fire growth will be governed by controls operating at coarser scales, such as weather or climate (top-down controls) [11], [13]. The changes of dominant controls as fire size increases are often called scale effects. Scale effects hypothesis are proposed as an explanation for the two hypotheses mentioned above, which argues that fire is determined by different controls operating at different scales. Supporting evidence for such scale effects comes from studies that evaluated controls on fire at several discrete spatial scales [14]–[17]. Currently, few studies have attempted to quantify the transition of landscape controls on fire across continuous spatial scales [12], [18].

According to the scale effects hypothesis, fire will reach a threshold value size at which its spead will be primarily determined by different spatial controls [13]; however, how to identify this threshold value, which switches the dominant controls, is not settled. Identifying the threshold value has important practical implications for fire or fuel management plans. For example, fire or fuel management may need to be altered on either side of the threshold value due to the change of dominant controls on fire; therefore, identifying such values is critical to designing effective management plans in areas where fire supression or fuel treatment are widely used, such as Northeast China [19], [20] or in the western US [21]. Because the relative importance of controls on fire across spatial scales may be nonlinear, identifying the threshold value requires a quantification of the relative importance of spatial controls on fire at continuous spatial scales, rather than several discrete spatial scales used by previous studies [13]–[16].

The relative influence of fuel and weather on fire may also be influenced by fuel composition (e.g., species composition and configuration) [6], [9], [10], [22]. Fuel characteristics influence the propagation and pattern of fire disturbance [23]. A heterogeneous fuel complex will greatly enhance the influence of fuel on fire. For example, Bergeron *et al*. [10] concluded that higher heterogeneity of fuel in the southern mixed-wood forest regulated fire spread more strongly than that in the northern boreal forests where coniferous trees dominated in western Quebec. Even in regions where climate exerted a dominant control on fires, fuel also strongly regulated the strength of correlations between climate and fires [22]. In contrast, a homogeneous fuel complex environment provides no choice for fire to burn preferentially, and therefore weather may correlate well with fire regimes irrespective of spatial scales [9]. The scale effects on the relative importance of fuel and weather on fire should therefore be region-specific due to differences in fuel characteristics.

Our main objective in this study was to test the scale effects of controls on fire spread across continuous spatial scales in a Chinese boreal forest landscape and to determine whether these scale effects are affected by fuel composition. Fuel composition in this study refers to proportional area of various fuel types in the “neighborhoods” of each individual fire patch (see *Methods: data* for details). We used fuel composition, weather at the time of fire occurrence, and local topography characteristic for each individual fire to quantify their relative importance on fire. We designed a “moving window” resampling technique that allowed us to quantify their relative importance on fire at continuous spatial scales. Specific research questions include: (1) did fire spread exhibit preferential burning among fuel type in this boreal forest landscape? (2) did scale effects hold true in explaining relative importance of fuel, topography, and weather across continuous spatial scales? and (3) can we find the threshold values for fire size that switch the dominant control of fire from bottom-up controls (i.e. fuel and topography) to top-down controls (i.e. weather)?

## Materials and Methods

### Study area

**Figure 1:**
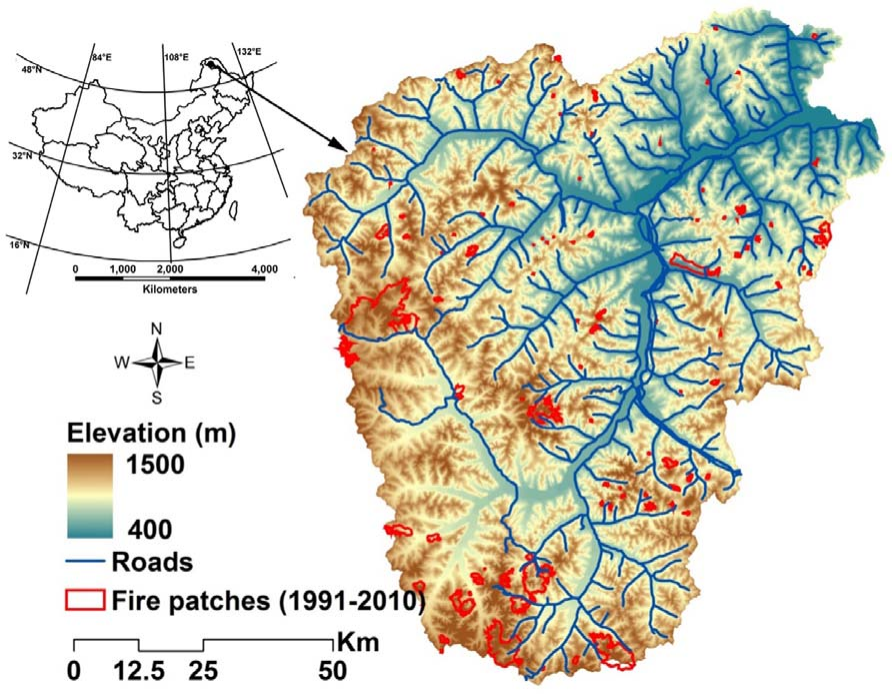
Study area with delineated fire patches from 1991 to 2010, roadway coverage, and digital elevation model.

Our study area was 937, 244 ha of boreal forest landscape in the Great Xing'an Mountains of Northeastern China (52°25′N 122°39′E to 51°14′N 124°21′E) (Figure 1). The area falls within the cool temperate zone with long and severe winters. The annual average temperature and precipitation are 4.7°C and ∼500 mm, respectively [24], [25]. Elevation varies gradually from 400 m in the northeast to 1500 m in the southwest. The vegetation is cool temperate coniferous forests, which are the southern extension of eastern Siberian boreal forests [25]. Larch (*Larix gmelini*), a widely distributed late successional coniferous species, is the most dominant tree species and can occupy a wide range of moisture gradient. Birch (*Betula platyphylla*), a widely distributed broadleaf species, intersperses with larch forest in xeric sites and can form pure stands in open spaces created by fire and harvest. Other species, such as Scotch pine (*Pinus sylvestris* var. *mongolica*), Koyama spruce (*Picea koraiensis*), willow (*Chosenia arbutifolia*), two species of aspen (*Populus davidiana* and *P. suaveolens*), and dwarf Siberian pine (*Pinus pumila*; occurs mostly in elevations >800 m) are interspersed with larch forest and have a small area of distribution (<2%).

Historical fire regime was characterized by frequent, low intensity surface fires mixed with infrequent stand-replacing fires [26]. Xu et al. [25] used fire scar data to conclude that the mean return interval was about 30 years for low intensity surface fires and 120 years for high intensity crown fires in pristine larch forests of this region. However, several decades of effective fire exclusion policy and extensive logging have dramatically changed fire regimes. Currently, fires are less frequent but more intense, with a much longer mean return interval than before these policies and activities [20], [27]. Clear-cutting combined with an altered fire regime has resulted in a fragmented forest landscape with simplified age structure and species composition. Since 1998, the Natural Forest Protection Project (NFPP) has been implemented to maintain forest sustainability. NFPP is similar to the zoning strategies proposed in North America (TRIAD or three zones) (MacLean et al. 2008), in which different management zones are established with different sets of objectives and priorities.

### Data

#### Dependent variable: fire data

We obtained a 20-year fire dataset for 146 fires from the Huzhong Forest Fire Prevention Agency (Huzhong, Heilongjiang, China, 165036) for 1991–2010 detailing fire origin location (recorded as *x*, *y* coordinate), size, date of occurrence, and extinction. We found that 89% of fires were caused by lightning, and most occurred in remote areas, either on high elevation sites or far from roads, which made fire suppression difficult.

We mapped all burned patches based on the fire dataset and Landsat Thematic Mapper (TM) imagery. The time series of TM images during August from 1990 to 2011 were downloaded from US Geological Service EROS Data Center (http://glovis.usgs.gov/). Based on the recorded fire origins and reflectance change of pre- and post-fire TM imageries, burned patch boundaries were visually identified in bands 2, 3, and 4 false-color composite images. We manually delineated 111 lightning burned patches from 1991 to 2010 (Figure 1). The other 35 fires recorded in the fire dataset could not be identified, either due to wrong location information or too small in size to be identified in the 30 m resolution TM imagery. The delineated burned patches were checked by local fire managers or revisited in the August 2011 field inventory when necessary.

Our analysis revealed that area calculated from manually delineated burned patches was not significantly different from the reported data; therefore, we are confident that our delineated burned patches captured the spatial characteristics of fires. Some larger burned patches may contain unburned islands with relatively small areas in valley bottoms. These small unburned islands within burned patches were difficult to map using TM imagery and therefore could not be delineated. The size of burned patches ranged from 1 to 8379 ha, with a median value of 31 ha. About 25% and 75% percentile were 7 and 100 ha, respectively. The burned patch size was used as the dependent variable in the analysis.

#### Explanatory variables: fuel, weather, and topography data for each individual fire. Fuel composition data

**Figure 2:**
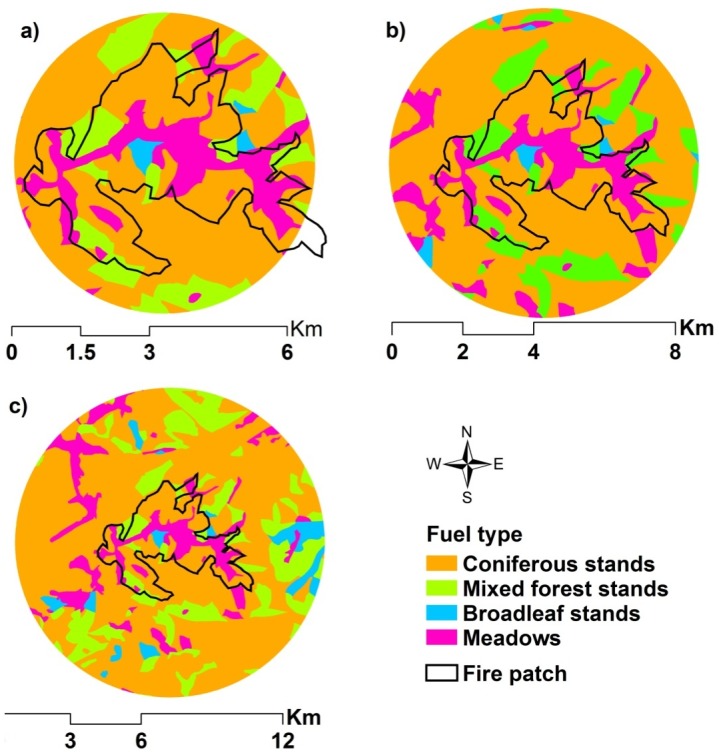
Schematic representations of a fires and its “neighborhood”. A “neighborhood” was defined as twice (a), 4 (b), and 8 (c) times the size of the actual fires patch, respectively.

We computed the composition of fuel for each individual fire within its neighborhood, which we defined as a circular area twice the size of the actual burned patch (Figure 2a). The composition of fuel within its neighborhood was considered as the locally available fuel conditions for the fire, a practice commonly used in previous studies [6], [9], [17]. The center of a neighborhood circle for a fire was the recorded origin location. As such, we assumed the fire starts from the origin and spreads equally in all directions without the influence of any other factors, such as fuel, weather, or topography. In this way, we can capture the effect of local fuel composition on fire spread. Although fuel composition calculated using this approach may be sensitive to fire ignition, we feel our fire ignition data are fairly accurate because most fires can be detected within several hours of occurrence due to strict fire prevention policy and effective fire monitoring networks. Because the fire prevention agency also requires the reporter to find evidence for cloud to ground striking point of all lightning fires, we are confident that the bias of available fuel type derived from the neighborhood will be limited.

We obtained forest stand maps of 1990 and 2000 produced from high resolution digital inventory data and updated every 10 years by the Forest Resource Management agency. The main utility of the forest stand map was to design management activities, such as harvesting. The forest stand map recorded relative percentage of canopy species and average age of dominant canopy species. Major understory species were also recorded, but quantitative information was lacking. For fires that occurred from 1991 to 2000, we intersected the 1990 forest stand map with the neighborhoods of each individual fire. For fires that occurred from 2001 to 2010, we intersected the 2000 forest stand map with neighborhoods of each individual fire.

We assigned intersected polygons within neighborhoods to one of four fuel types: coniferous, mixed, broadleaf, and meadow and others. The first three fuel types are based on the polygon's dominant tree species. If a polygon was mainly composed of coniferous or broadleaf trees (relative percentage >70%), the polygon was assigned to a coniferous or broadleaf fuel type. Otherwise, the polygon was assigned to a mixed forest fuel type. Fuel type “meadow and others” includes all non-forested areas, predominantly grassland on the newly burned or harvested areas and wetlands (hereafter: meadows). Our statistics revealed that the median size for a stand (fuel type) is 23.1 ha; the 25th and 75th percentile sizes were 12.8 and 34.7 ha, respectively. Further analysis indicated that coniferous forest fuel type constituted 54.4% of total fuel type; the 25th and 75th percentile sizes were 12.5 and 33.9 ha, respectively. Mixed forest fuel type constituted 22.6% of total fuel type; the 25th and 75th percentile sizes were 15.3 and 36.7 ha, respectively. Broadleaf forest fuel type constituted 13.6% of total fuel type; the 25th and 75th percentile sizes were 14.2 and 36.4 ha, respectively. Meadows fuel type constituted 5% of total fuel type; the 25th and 75th percentile sizes were 5 and 25.3 ha, respectively (Figure S1).

The fuel composition within a neighborhood was calculated as the percentage in area of each of the fuel types. Following this procedure, a 4-dimensional vector was constructed with elements proportional to each fuel type within the neighborhood. We let A*_i_* be the total area of neighborhood of the *i*th fire, w*_i,j_* be the area of fuel type *j* in the *i*th fire, and the vector of proportions x_i_ is calculated as:
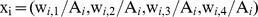
with elements being the proportional areas of the coniferous, mixed, broadleaf stand, and meadows, respectively. The calculation of fuel composition data in this study is similar to Cumming [6] and Podur & Martell [9].

For each fire, we also intersected burned patch with the forest stand map to calculate the burned fuel composition within each individual fire, following the procedure for fuel composition within its neighborhoods. The fuel composition within a burned patch (which can be considered as observed burned fuel composition) was compared with fuel composition within its neighborhood (which can be considered as expected burned fuel composition) to determine whether the burn exhibited a preferential fuel type.

#### Fire weather data

We used antecedent and post-ignition fire weather data for each corresponding fire in the analysis. Six daily fire weather indices were computed based on Van Wagner [28]: Fine Fuel Moisture Code (FFMC), Duff Moisture Code (DMC), Drought Code (DC), Initial Spread Index (ISI), Buildup Index (BUI), and Fire Weather Index (FWI). The first three indices are fuel moisture codes, which are numerical ratings of the moisture content of different fuel layers on the forest floor, highly correlated to fire occurrence [29]. The remaining three components are fire behavior indices, which represent the rate of fire spread and the frontal fire intensity; their values rise as the fire danger increases.

Calculation of fire weather indices is based on consecutive daily observations of temperature, relative humidity, wind speed, and 24-hour rainfall. Daily meteorological data for our study area were obtained from National Centers for Environmental Prediction (NECP) reanalysis-2 data (http://www.esrl.noaa.gov/psd/, accessed 7 October 2011) for 1991 to 2010 because daily meteorological data for our study area are scarce or incomplete. The NECP reanalysis data have a spatial resolution of 1.875×1.92 degrees. We extracted daily meteorological data from a grid cell that falls within our study area to calculate fire weather indices. Fire weather indices for each individual fire were calculated as the average value of 30 daily codes preceding fire occurrence to date and daily codes during the fire initiation and extinction. Daily codes for the 30 days preceding fire were used because, based on preliminary analysis (data not shown), they are more strongly related to fire size than the 15 or 45 days preceding fire.

In this study, fire weather anomalies, which were calculated as the difference between indices value for each individual fire and 20-year (1991–2010) average, were used to quantify its effect on fire size. Fire weather anomalies captured the weather conditions at the actual time of fire occurrence. Preliminary analysis indicated that fire size was positively correlated to fire weather anomalies, except for DC (Figure S2), possibly because DC is an indicator of seasonal drought and therefore cannot capture the short term effects of weather on fire. DC was therefore removed from the subsequent analysis.

#### Topography data

Local topography data used in this study included elevation, aspect, and slope within in the neighborhood of each fire. A digital elevation model (DEM) was generated from 1∶100 000 contour lines map at 10 m intervals. Slope and aspect surfaces were derived from the DEM. Aspect was further converted into potential solar radiation (Poten_rad) using the formula described by [30]:
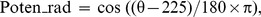
where θ is the aspect derived from Arc/Info “aspect” function, which ranged from 0 to 360. The Poten_rad ranged from −1 to 1, with higher values indicating higher potential solar radiation. Local topography data were calculated as mean values in the neighborhood because previous studies have suggested that topographic characteristics within a neighborhood may hold more relevant information to controls on fire regimes [14], [17]. Analysis indicated that local topography characteristic data were normally distributed, with mean values of 16° slope, 900 m elevation, and 0 aspect (Figure S3).

#### Fire suppression data

According to fire management policy, all fire should be aggressively attacked irrespective of their locations in this area [19]. To account for fire suppression effects on fire spread, we used distance to nearest road (Dis_Rd) as a simple indicator of fire suppression efficiency. A shorter distance to road allows easier access to burned patches, thus may render higher fire suppression efficiency. Dis_Rd was calculated using Arc/Info “distance” function.

### Regression modeling

We constructed regression models of fire size using boosted regression trees (BRTs) for the explanatory variables (Table 1). The BRT method combines the strengths of two algorithms, regression trees and boosting, and is suitable for ecological analyses because of its flexibility in modeling complex nonlinear relationships, analyzing different data types, relatively transparent approach, and interpretability of output in describing relationships between dependent and independent variables [31]–[33]. The BRT method fits the best possible model to the data structure through an iterative partitioning approach of regression trees, but it reduces predictive error by “boosting” initial models with additional, sequential trees that model the residuals in randomized subsets of the data [31], [32]. An a priori model specification or test of hypothesis was therefore not required, and because BRTs can accommodate virtually any data distribution, no transformations were required. Recent comparative analyses of ecological datasets suggested BRT models often outperform alternative statistical approaches in terms of predictive accuracy and interpretability [16], [34].

**Table 1:** Dependent and exploratory variables used to assess the relative importance of topography, fuel, and fire weather on fire spread in the boreal forest of Northeast China from 1990 to 2010.

Variable name	description	mean ± sd
Fire (dependent)	Patch size for each individual fire	248±898 (ha)
Fuel		
Conif_Pct	Percentage of coniferous forest available for burn within each neighborhood circle	68.6±31.6
Mixed_Pct	Percentage of mixed forest available for burn within each neighborhood circle	14.9±23.1
Broad_Pct	Percentage of broadleaf forest available for burn within each neighborhood circle	9.1±19.7
Meadows_Pct	Percentage of other fuels available for burn within each neighborhood circle	8.0±15.6
Fuel_age	Stand age for forest fuel (e.g., Conif_Pct, Mixed_Pct, Broadl_Pct)	84.2±27.3 (yrs)
Fire weather anomalies		
FFMC	The Fine Fuel Moisture Code (FFMC) is a numeric rating of the moisture content of litter and other cured fine fuels. This code is an indicator of the relative ease of ignition and the flammability of fine fuel.	9.91±8.66
DMC	The Duff Moisture Code (DMC) is a numeric rating of the average moisture content of loosely compacted organic layers of moderate depth. This code gives an indication of fuel consumption in moderate duff layers and medium-size woody material.	9.46±7.70
ISI	The Initial Spread Index (ISI) is a numeric rating of the expected rate of fire spread. It combines the effects of wind and the FFMC on rate of spread without the influence of variable quantities of fuel.	0.93±1.18
BUI	The Buildup Index is a numeric rating of the total amount of fuel available for combustion. It combines the DMC and the DC	13.09±8.62
FWI	The Fire Weather Index is a numeric rating of fire intensity. It combines the Initial Spread Index and the Buildup Index. It is suitable as a general index of forest fire danger.	2.35±2.60
Topography		
Elev	Mean elevation within neighborhood circle for each fire	911±150 (m)
Aspect	Mean aspect within neighborhood circle for each fire	−0.059±0.482
Slope	Mean slope within neighborhood circle for each fire	11.45±4.60 (degrees)
Fire suppression		
Dis_Rd	Distance to nearest road for each fire	2345.2±3587 (m)

We used fire size as the dependent variable with respect to explanatory variables and implemented the BRT analysis using “gbm” package version 1.5.7 in R 2.13 [35]. BRT involves subsampling and bagging of the dataset to improve model fitting and introduce randomness. To limit the stochasticity in model outcomes caused by the subsampling and bagging, we created an ensemble of 50 BRT models for each model run and then averaged the results. In this study, we used the following parameters when fitting regression trees: bag fraction = 0.5, shrinkage rate = 0.005 and number of trees = 300. The BRT method reports the relative importance of each explanatory variable by averaging the number of times it is selected as a tree node over all trees and the squared improvements resulting from these nodes.

To reduce the potential collinearity in explanatory variables, we conducted a Pearson correlation analysis between all explanatory variables. A Pearson rank correlation matrix showed a strong pairwise correlation between fire weather indices (r>0.8) but not for other variables (r<0.4); therefore, we performed a principal component analysis to reduce the collinearity between fire weather indexes. We selected the first three axes for principle component analysis because they explained more than 98% of the information of fire weather indices. The first three axes were therefore used as fire weather data instead of the five fire weather indices in the subsequent analysis.

### Analysis of data

#### Preferential burning analysis

To test whether fires exhibit preferential burning for a fuel type, we compared the log-ratios of the proportion of observed burned fuel composition with expected burned fuel composition using a pairwise student's t test to determine whether the compositions are significantly different from one another at a given confidence level. Detailed procedures can be found at Cumming [6] and Podur & Martell [9].

#### Quantifying the relative importance of fuel, topography, and weather on fire across the continuous spatial scales

**Figure 3:**
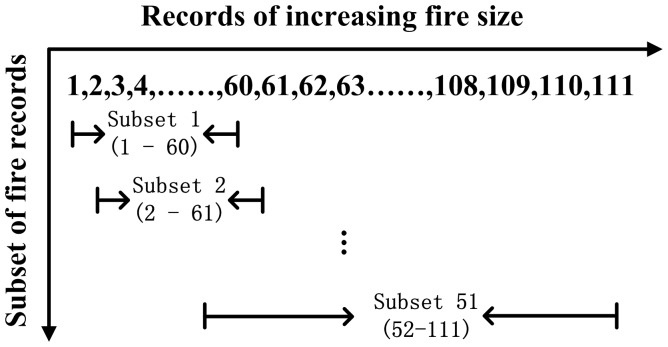
Schematic representations of resampling procedure of subset from dataset.

We designed a “moving window” resampling approach to quantify the relative importance of fuel, topography, and weather on fire across the continuous spatial scales. We first reorganized the fire dataset by fire size in ascending order. We then subset 60 fires each time from the reorganized dataset by size, similar to the “one dimension moving window” approach (Figure 3). This resampling procedure produced 51 subsets of 60 fires, with a mean fire size from 11 to 445 ha. We selected 60 fires to make a balance between sample size for performing BRT analysis (60 fires) and plotting the relative importance of controls to the fire size (51 subsets). Finally, we used the BRT method to quantify the relative importance of each individual variable on fire size using the 51 subsets. Dis_Rd was included as an independent variable in the BRT analysis but was not included in the result presentation because its relative importance is not the focus of the current study. We grouped the relative importance of each variable into fuel, topography, and weather. The relative contribution of fuel, topography, and weather, expressed as a percentage, was plotted for mean fire size to show their transition across the continuous spatial scales.

Based on the relative importance of fuel, topography, and weather on fire across the continuous spatial scales, we identified the threshold of fire size that switches the dominant control of fire from bottom-up controls (e.g., fuel and topography) to top-down controls (e.g., weather) by comparing the point (fire size) at which the relative importance of weather outweighs the relative importance of fuel. Because fire size was positively correlated to fire weather anomalies (Figure S2), we could also identify the threshold for fire weather anomalies that switches the dominant controls of fire. We identified the threshold for fire weather anomalies because it can easily be calculated based on local weather station data and also have predictive value for fire size.

#### Effects of fuel composition on the continuous transition of various influences on fire

To test the effects of fuel composition on the influence of spatial controls on fire, we increased the size of the neighborhood for each fire to 4 (Figure 2b) and 8 (Figure 2c) times that of the actual fires and recomputed the expected fuel composition. Enlarging the neighborhoods introduced different fuel types unequally by changing the expected fuel composition in the neighborhoods. Our analysis indicated that enlarging the neighborhoods introduced more fuel types of mixed stands and fewer fuel types of coniferous, broadleaf and meadows (Figure S4). Enlarging the neighborhoods also ensured that the most complex burned patches were completely incorporated into the neighborhoods. We used the fuel composition data and recomputed the relative importance of fuel, topography, and weather on fire across the spatial scales as described in the previous section.

## Results

Analysis indicated that fire exhibited preferential burning patterns among different fuel types (p<0.05) (Table 2). The observed annual burned rate was significantly lower than expected in coniferous stands (p<0.05), while meadows showed an opposite trend (p<0.05). Mixed and broadleaf stands showed a slightly lower observed burned rate than expected but did not reach a significant level (p>0.05). To further examine whether preferential burning varied with fire size, we divided the fire dataset into three subsamples by equal quintiles. Our first subsample included all fires <10 ha, and the second subsamples included all fires between 10 and 67 ha. We found no preferential burning for any of the four fuel components for the first two subsamples. Our third subsample included all fires >67 ha, and we found preferential burning among different fuel types similar to that for all fires.

**Table 2:** local-scale fire preferential burning, summarized as burned area, annual burned rate and mean fire rotation period for each fuel type.

Fuel type	Burned area (ha)(20 years)		Annual burned rate (×10^4^) (<$>\raster(90%)="rg1"<$>)		Fire Rotation Period (years)		*p* value
	Expected	Observed	Expected	Observed	Expected	Observed	
coniferous	21349.81	20476.9	20.90	20.05	478	497	<0.05
Mixed	2400.937	2188.405	5.67	5.17	1763	1934	>0.05
Broadleaf	1072.959	1026.245	4.21	4.03	2375	2484	>0.05
Meadow and others	2722.021	3853.723	15.45	21.88	647	457	<0.05
total	27545.72	14.68	681	<0.05			

Notes: Burned area is summarized as total area burned from 1991–2010 (20 years). Annual burned rate is proportion of area burned per year. Fire returned interval is the average interval between fires at a given site. Expected values were calculated from the “neighborhoods” of fires; Observed values were calculated from the actual burned patch. P value was calculated whether there is a significant difference between expected and observed value. <$>\raster(90%)="rg1"<$> stands for per 10,000.

**Figure 4:**
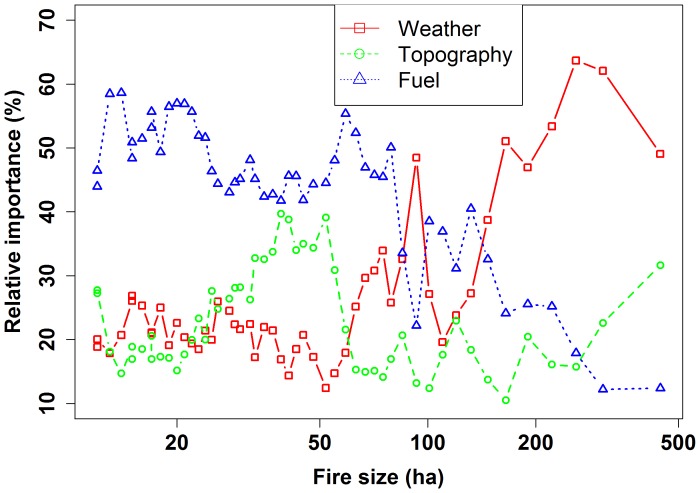
Relative influence of fuel, topography, and fire weather with increasing fire size. X-axes are plotted on a log_10_ scale. Data was plotted based on the average value from 3 fuel composition data.

**Figure 5:**
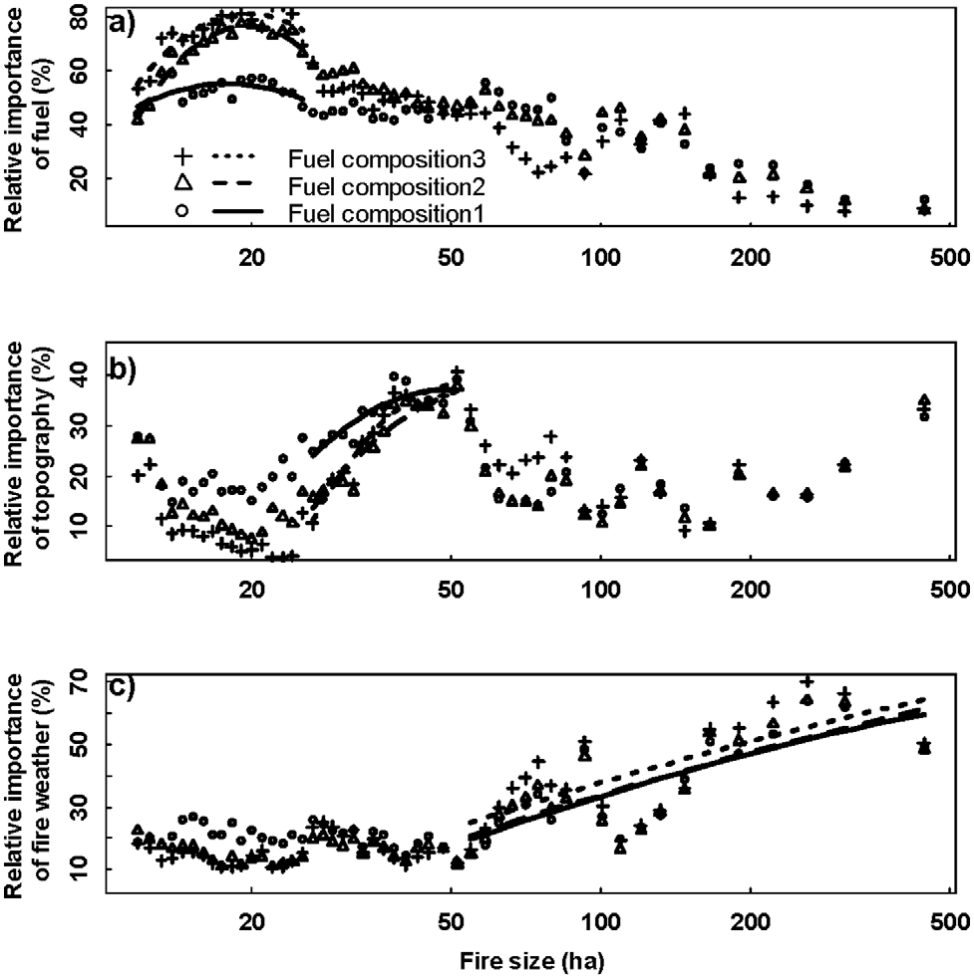
Relative influence of a) fuel, b) topography, and c) weather on fire size. X-axes are plotted on a log_10_ scale. Fuel composition 1, 2, and 3 stands for expected proportion of fuel types within “neighborhoods” of 2, 4, and 8 times of actual fires.

The prediction error of BRT analysis varied between 14 and 18%. Generally, the prediction error did not change dramatically with fire size (Figure S5), suggesting a relatively consistent prediction power of BRT analysis. The BRT analysis indicated that the relative importance of fuel, weather, and topography changed continuously with fire size. Fuel played a dominant role in affecting small fires (<75 ha in our case) (Figure 4). Fuel, topography, and weather exerted relatively comparable influence on medium fires (76–150 ha in our case) (Figure 4). Fire weather had a dominant role when the fire size was >150 ha (Figure 4). Afterward, the larger fire size, the more important the influence of fire weather. Generally, fire weather will gain more relative influence from small to large scale, while fuel has an opposite trend. The relative importance of topography fluctuated throughout spatial scales but was generally below 30% (Figure 4 and 5). A difference in fuel composition influenced the relative importance of fuel, topography, and weather but did not change the general pattern of their relative importance (Figure 5). Generally, the effects of changing fuel composition on the relative importance of fuel, topography, and weather are prominent for small fires but indistinguishable for large fires (Figure 5).

Generally, most fires occurred under favorable fire weather conditions (fire weather anomalies >0). The higher the value of fire weather anomalies, the larger the fire size. The BRT analysis indicated that weather was the dominant control when fire size was >150 ha, which can be translated into 70th percentiles of fire weather anomalies; therefore, a fire size larger than 150 ha or 70th percentiles of fire weather anomalies switches the dominant controls on fire from bottom-up controls (e.g., fuel and topography) to top-down controls (e.g., weather) for our study area.

## Discussion

### Preferential burning among fuel types

Our results suggested that fire burned fuel types selectively in this Chinese boreal forest landscape, although the selectivity is low. Our results therefore support studies finding that fire burns preferentially among fuel types in many other boreal forests [6], [36], but contradict others [9]; however, even studies that support preferential burning among fuel types disagree on which fuel types are more fire prone [6], [37], [38]. For example, Cumming [6] used compositional analysis methods to assess the extent that fuel composition burned by 48 fires between 1961 and 1997 in a boreal mixed-wood region of Alberta differed from the composition of available fuel, and found conifer-dominated stands are 3–10 times more prone to burning than deciduous stands. However, Larsen [37] estimated the time since last fire at 166 sample patches and found higher fire susceptibility in aspen stands compared to white spruce and black spruce stands. We believe that whether fire exhibited preferential burning or which fuel type is more susceptible to fire will be dependent on fuel characteristics being studied.

Fire selected more strongly for meadows than expected, because, we speculate, fine fuels are easily ignited and greatly accelerate the rate of spread in this vegetation type [39]. Furthermore, a previous study found that increased availability of flammable fine fuels has positive effects on lightning fire frequency, and therefore contributed more to burned area [40]. Coniferous stands were significantly less fire prone in our analysis which was unexpected because many studies have indicated that coniferous stands tend to contain more flammable fuel conditions [6], [38] and have high initiation probability [8]. Our results are also predictable, however, because intensive forest thinning has greatly reduced the surface and ladder fuels in this Chinese boreal landscape, which may lead to significantly lower fuel loading than that in the North American boreal forest [41]. The low burned rate in coniferous stands in our study area may also result from interaction with other environmental factors. Coniferous stands often dominate wet, cool north slopes, where fuel moisture content is generally higher, resulting in fewer fires. Our previous study also revealed that fewer fires occurred than expected in this region [42]. For mixed and broadleaf stands, there were fewer fires than expected, and these did not reach a significant level. These two types of forest stands usually inhabit xeric south slopes, where fuel loading is generally lower due to higher decomposition rate [43]. Furthermore, deciduous litter is less flammable, which also contributed to a lower burn rate [38], resulting in fewer fires in these two forest types.

### Relative importance of fuel, topography, and weather on fire spread

Our results suggested that weather was the dominant control on larger fires but not small fires, possibly because larger fires were generally more severe, and previous studies have indicated that fire severity is positively related to fire size in boreal forests [44], [45]. Larger, more severe fires consume different fuel types without preference and are therefore more strongly related to weather conditions, but for smaller and less severe fires, fuel type played a more important role in limiting fire size [46]. Generally, the top-down controls gained more influence, while the bottom-up controls such as fuel gained less influence when the fire size becomes larger; therefore, the scaling effect of spatial controls on fire holds true in this Chinese boreal forest landscape, which is consistent with other studies [14]–[17].

Our results found that the relative importance of topography on fire was rather persistent when fire size is <30 ha and then fluctuated dramatically with increasing fire size. Topography had a minimum influence on small fires (fire size <30 ha). Most small fires burn within a single fuel type (see our *Limitations* section below), which is generally contained within similar topographic characteristics; therefore, the relative importance of fuel on fire spread overwhelmed the effects of topography (Figure 4). However, the influence of topography, through its interaction with fuel and climate, on fire spread gradually emerges when fires became larger. For example, at fine scales, topography only influences fire spread through its regulation on local fuel characteristic, such as moisture, continuity, and loading. At large scales, the topography determines fire spread by regulating vegetation structure and local weather conditions. The effects of topography may therefore have a varied influence on fire spread across spatial scales.

Changing fuel composition had a larger effect on the relative importance of spatial controls for smaller fires (Figure 5), possibly because smaller fires were usually dominated by a single large fuel type while larger fires were usually dominated by more fuel types. Therefore, enlarging the neighborhood may have a larger effect on fuel composition for small fires, but only a trivial effect on large fires. We did not examine the effects of multiple neighborhood sizes for the topographic variables on the relative importance of spatial controls on fire size; however, we do not believe enlarging the neighborhood will have a significant effect on the results because (1) topography is relatively flat in our study area, and changing neighborhood size may not alter the local topographic characteristics; (2) local topographic characteristic data were normally distributed, and changing neighborhood size may not alter the mean value of topographic variables.

Humans can exert a complex effect on fire regime in this human-dominated boreal landscape, which may affect the scale behavior of fire. Humans tend to increase ignition density by roughly 50% of total fires [42], but they also contribute to a decrease in fire size through suppression and management activities. Consequently, humans are highly likely to have a nonlinear effect on fire regime, which complicates prediction of human effects on scaling behavior of controls. We can expect human activities to increase the influence of weather on fire size, however, because forest management tends to homogenize the surface fuel loads [41] and therefore tends to diminish the effects of fuel on fire. Such conclusions require more support from both empirical and simulation studies, however.

### Results implications

Landscape fuel management via fuel breaks is a major fire management strategy, based on the assumption that fires will spread more slowly and be less intense in the fuel breaks, and therefore will be more easily suppressed [21]. Based on our analysis, this assumption may be valid if fire size is below a certain critical threshold and when fuel is the main limiting factor for fire spread. When fire size reached that threshold value or above, however, fires were driven primarily by weather, and the fuel break will be less effective. This threshold value is different among forest ecosystems and can be potentially determined by transitions of landscape controls on fires using a moving window resampling technique. For forest landscape fire modellers, our results also have practical implications. For example, when a disturbed area is the focus of simulation, weather conditions should be carefully parameterized because a few large fires accounted for the majority of burned area [47]. If ignition density is the focus, fuel characteristics and topography should be examined.

Wildfire is an ecological disturbance that tends to be governed by cross-scale interaction [13], which is critical to understanding the scale behaviour of wildfire. Recognizing the dominant controls on fire spread change across a spatial extent is the first step to forecast its behaviour by focusing on the importance of scale and process. Peters et al. [13] proposed a general framework for understanding the occurrence and consequence of fire across spatial and temporal scales. The core of their framework is to identify the threshold that can trigger cross-scale interaction, but identifying that threshold before it occurs is a critical challenge. Our approach provided a potential alternative to identify the threshold that can trigger cross-scale processes.

### Limitations

#### Data quality

In this study, we did not delineate unburned islands within the fire perimeter. Although unburned islands can be prevalent in larger fires of North America boreal forests [44], they are not in our study area because (1) the unburned islands (also called fire refugia) may be prevalent in moist sites, such as valley bottoms, but these constitute a small proportion (<2%) in this area; (2) the fire size was generally small, so that the resulting bias may be limited.

Our fuel type data were derived from the official forest stand maps, which were the most accurate and consistent vegetation data to date. Our meadows category mainly contains grass, shrub, and wetland, a fuel type shown by our preferential burning analysis to favor fire spread. This seems somewhat counterintuitive for wetlands, because studies have indicated that wetlands may act as fuel breaks. However, in our study area, wetland is often confined within the bottomland where permafrost exists. In summer, wetlands may block fire spread because of higher fuel moisture and abundant water, but in spring (March to May), the main fire season with little precipitation, the brown vegetation (mainly *Carex tristachya* and *Betula fruticosa Pall*., which provided ample fine fuel) actually favor fire spread (CM Zhang, Huzhong forest bureau, pers. comm.). Although the meadows category constitutes a small area, it is critical to fire management because of its flammability and fast rate of spread. We did not include a non-flammable fuel type (e.g., water body) in the analysis because that category constitutes a negligible area (<1%), and our visual inspection did not find any non-flammable areas within burned patches or neighborhoods. Due to limited data, we were not able to relate overstory fuel type to soil carbon and ground cover (only species were recorded in the forest stand map; no coverage or proportion data were included), although overstory constitutes a large portion of the combusted fuel.

The results of our preferential burning analysis were not significantly different among fuel types when fire size was <67 ha because most small fires burn within a single fuel type. For example, the dominant fuel type often constituted more than 98% of the burned area and neighborhoods of small fires. At this stage (within-patch stage; see Figure 1 in [13]), local processes, such as chemistry and amount of fuel within a patch, determined the fire spread. Under these conditions, our compositional analysis may fail to detect significant differences between observed and expected proportion of various fuel types for small fires. With the increase in fire size, the next stage (among patches spread stages; see Figure 1 in [13]) was reached, and the spread of fire was controlled by fuel load and connectivity of fuel types. The difference between observed and expected proportion of various fuel type became more evident and could be detected by the compositional analysis. As a fire grows, a threshold will be reached where fire spread is no longer determined by local fuel conditions. At this stage (feedbacks stage; see Figure 1 in [13]), the fire climate will probably overwhelm local processes (e.g., fuel characteristics) as the dominant factor of fire spread. Furthermore, fires at this stage can produce enough heat to generate their own weather, allowing even more rapid fire spread. Our compositional analysis therefore focused on analyzing the preferential burning of various fuel types across scales, in contrast to BRT analysis that focused on identifying the dominant scaling behavior. Note, however, that the inaccuracy of fuel type data, which can affect the performance of compositional analysis, and prediction error of BRT analysis may also affect the comparison. Nevertheless, these two analyses are complementary and useful for fuel and fire management.

Fire size is the result of the interaction of fuel, topography, and weather at multiple spatial scales; however, fire may also affect fuel connectivity and configuration, which may in turn influence the next fire spread. This interactive effect between fires is sometime termed as ecological memory or self-regulation [48] of disturbance events. For our analysis, this effect may compound the influence of spatial controls on fire spread; therefore, the dependent variable (fire size) should be considered as a quasi-dependent variable to some degree. This effect is unlikely in our analysis, however, because of the long fire return interval (>500 yr) [20], [27] and short fire data period (20 yr). In our BRT analysis, fire size was treated as a response variable that can be explained by various spatial controls. We then plotted the relative importance of various controls across spatial scales, measured by fire size; therefore, fire size acted individually for the BRT analysis and the plotting. From this information, we could determine the threshold of fire size that switched the dominant controls of fire spread.

#### Analytical method

The “moving windows” resampling technique is easy to use, and the results are straightforward; however, this technique requires a large dataset to yield a reliable outcome because bagging and regression are involved in the process. Without extensive data, analyses may show a wide variation due to stochasticity (Figure 4). Moreover, various methods to measure the dependent variable will also affect the threshold. For example, when we used median fire size as the dependent variable, the threshold for fire size was about 100 ha because of decaying distribution of each sample, but this did not affect the scale behavior of relative importance of each control across continuous spatial scale. Further, the width of the moving windows may significantly affect threshold identification because the observations influence both BRT regression processes and the value of the dependent variable; therefore, the threshold derived from this analytical method relied heavily on the sampling technique and methods to determine the dependent variable. Nevertheless, our proposed method provides a potential alternative to understanding the threshold behavior of wildfires across space. This information is important for forecasting ecosystem dynamics influenced by spatial nonlinear phenomenon [13].

## Conclusions

Fire spread is a spatially contiguous process regulated by multiple controls, such as fuel, topography, and weather. But the relative importance of these controls on fire across continuous spatial scales has not been clearly understood. Our analysis of scaling behavior and the detection of thresholds in fire spread was achieved using BRT analysis and the moving window resampling technique. Our method used commonly available datasets describing fire size and spatial controls. The availability of these data, together with an easily implemented approach, can be applied to identify scale thresholds in wildfire regimes. Our analysis indicated that (1) with increasing fire size, the dominant control of fire switched from bottom-up controls (fuel and topography) to top-down controls (weather); and (2) a threshold for fire size will be reached, above which fires are driven primarily by weather. The threshold, which may be ecosystem-specific, can be identified using the “moving windows” method introduced in this study.

## Supporting Information

Figure S1 **Patch size distribution for a) coniferous; b) mixed; c) broadleaf; and d) meadows within fire scars.**
(TIF)

Figure S2 **The linear relationship between fire size and anomaly of a) ISI, b)BUI, c)FWI, d)FFMC, and e) DMC.** The y axis (fire size) was logarithmic transformed.
(TIF)

Figure S3 **Histogram distribution of a) slope; b) elevation; and c) aspect within fire scars.**
(TIF)

Figure S4 **Percentage of a) mixed, b) coniferous, c)broadleaf, and d) meadows within a neighborhood with different size.** Fuel composistion 1, 2 and 3 was computed in neighborhood with size of 2, 4, and 8 times of actual fire. The x axis (fire size) was logarithmic transformed.
(TIF)

Figure S5 **Prediction error of BRT with different fire size. The x axis (fire size) was logarithmic transformed.** The prediction error was calculated as follows: Prediction error = (predicted.value - observed.value) ×100/observed.value.
(TIF)

## References

[1] Bowman DMJS, Balch JK, Artaxo P, Bond WJ, Carlson JM, et al (2009) Fire in the Earth System. Science 324: 481–484. 19390038 10.1126/science.1163886

[2] Bond W, Keeley J (2005) Fire as a global herbivore': the ecology and evolution of flammable ecosystems. Trends Ecol Evol 20: 387–394. 16701401 10.1016/j.tree.2005.04.025

[3] Bond W, Woodward F, Midgley G (2005) The global distribution of ecosystems in a world without fire. New Phytol 165: 525–538. 15720663 10.1111/j.1469-8137.2004.01252.x

[4] Keeley JE, Pausas JG, Rundel PW, Bond WJ, Bradstock RA (2011) Fire as an evolutionary pressure shaping plant traits. Trends Plant Sci 16: 406–411. 21571573 10.1016/j.tplants.2011.04.002

[5] Bessie W, Johnson E (1995) The relative importance of fuels and weather on fire behavior in subalpine forests. Ecology 76: 747–762.

[6] Cumming S (2001) Forest type and wildfire in the Alberta boreal mixedwood: what do fires burn? Ecol Appl 11: 97–110.

[7] Schoennagel T, Veblen TT, Romme WH (2004) The interaction of fire, fuels, and climate across Rocky Mountain forests. BioScience 54: 661–676.

[8] Krawchuk M, Cumming S, Flannigan M, Wein R (2006) Biotic and abiotic regulation of lightning fire initiation in the mixedwood boreal forest. 87: 458–468. 10.1890/05-1021 16637370

[9] Podur JJ, Martell DL (2009) The influence of weather and fuel type on the fuel composition of the area burned by forest fires in Ontario, 1996–2006. Ecol Appl 19: 1246–1252. 19688931 10.1890/08-0790.1

[10] Bergeron Y, Gauthier S, Flannigan M, Kafka V (2004) Fire regimes at the transition between mixedwood and coniferous boreal forest in northwestern Quebec. Ecology 85: 1916–1932.

[11] Falk DA, Heyerdahl EK, Brown PM, Farris C, Fulé PZ, et al (2011) Multi-scale controls of historical forest-fire regimes: new insights from fire-scar networks. Frontiers Ecol Environ 9: 446–454.

[12] Slocum MG, Beckage B, Platt WJ, Orzell SL, Taylor W (2010) Effect of climate on wildfire size: a cross-scale analysis. Ecosystems 13: 828–840.

[13] Peters DPC, Pielke Sr RA, Bestelmeyer BT, Allen CD, Munson-McGee S, et al (2004) Cross-scale interactions, nonlinearities, and forecasting catastrophic events. PNAS 101: 15130–15135. 15469919 10.1073/pnas.0403822101 PMC523446

[14] Cyr D, Gauthier S, Bergeron Y (2007) Scale-dependent determinants of heterogeneity in fire frequency in a coniferous boreal forest of eastern Canada. Landscape Ecol 22: 1325–1339.

[15] Parisien M-A, Parks SA, Krawchuk MA, Flannigan MD, Bowman LM, et al (2011) Scale-dependent controls on the area burned in the boreal forest of Canada, 1980–2005. Ecol Appl 21: 789–805. 21639045 10.1890/10-0326.1

[16] Parisien M-A, Moritz MA (2009) Environmental controls on the distribution of wildfire at multiple spatial scales. Ecol Monogr 79: 127–154.

[17] Parks SA, Parisien MA, Miller C (2011) Multi-scale evaluation of the environmental controls on burn probability in a southern Sierra Nevada landscape. Int J Wildland Fire 20: 815–828.

[18] McKenzie D, Kennedy MC (2011) Power-law behavior reveals phase transitions in landscape controls of fire regimes. Nature Preced http://www.ncbi.nlm.nih.gov/pubmed/22395617 10.1038/ncomms1731 22395617

[19] Chang Y, He HS, Bishop I, Hu YM, Bu RC, et al (2007) Long-term forest landscape responses to fire exclusion in the Great Xing'an Mountains, China. Int J Wildland Fire 16: 34–44.

[20] Liu Z, He HS, Chang Y, Hu Y (2010) Analyzing the effectiveness of alternative fuel reductions of a forested landscape in Northeastern China. For Ecol Manage 259: 1255–1261.

[21] Agee JK, Skinner CN (2005) Basic principles of forest fuel reduction treatments. For Ecol Manage 211: 83–96.

[22] Littell JS, McKenzie D, Peterson DL, Westerling AL (2009) Climate and wildfire area burned in western US ecoprovinces, 1916–2003. Ecol Appl 19: 1003–1021. 19544740 10.1890/07-1183.1

[23] Hargrove W, Gardner R, Turner M, Romme W, Despain D (2000) Simulating fire patterns in heterogeneous landscapes. Ecol Model 135: 243–263.

[24] Zhou YL (1991) Vegetation in Great Xing' an Mountains of China. Beijing: Science Press. 1–216 p.

[25] Xu HC (1998) Forest in Great Xing' an Mountains of China. Beijing: Science Press. 1–231 p.

[26] Xu CH, Li ZD, Qiu Y (1997) Fire disturbance history in virgin forest in northern region of daxinganling mountains. Acta Ecologica Sinica 17: 3–9.

[27] Liu Z, He HS, Yang J (2012) Emulating natural fire effects using harvesting in an eastern boreal forest landscape of northeast China. J Veg Sci 23: 782–795.

[28] Van Wagner C (1987) Development and structure of the Canadian forest fire weather index system: Canadian Forestry Service, Petawawa National Forestry Institute, Forestry technical Report FTR-35,. Chalk River, Ontario 36.

[29] Wotton BM, Nock CA, Flannigan MD (2010) Forest fire occurrence and climate change in Canada. Int J Wildland Fire 19: 253–271.

[30] Franklin J, McCullough P, Gray C (2000) Terrain variables used for predictive mapping of vegetation communities in southern California,In: Wilson, J.P., Gallant, J.C. (Eds.), Terrain Analysis: Principles and Applications. Wiley, New York.

[31] Elith J, Leathwick J, Hastie T (2008) A working guide to boosted regression trees. J Anim Ecol 77: 802–813. 18397250 10.1111/j.1365-2656.2008.01390.x

[32] De'Ath G (2007) Boosted trees for ecological modeling and prediction. Ecology 88: 243–251. 17489472 10.1890/0012-9658(2007)88[243:btfema]2.0.co;2

[33] Prasad AM, Iverson LR, Liaw A (2006) Newer classification and regression tree techniques: bagging and random forests for ecological prediction. Ecosystems 9: 181–199.

[34] Elith J, Graham* CH, Anderson RP, Dudik M, Ferrier S, et al (2006) Novel methods improve prediction of species' distributions from occurrence data. Ecography 29: 129–151.

[35] Ridgeway G (2006) Generalized boosted regression models. Documentation on the R Package ‘gbm’ version 1·: 5–7.

[36] Hély C, Fortin CMJ, Anderson KR, Bergeron Y (2011) Landscape composition influences local pattern of fire size in the eastern Canadian boreal forest: role of weather and landscape mosaic on fire size distribution in mixedwood boreal forest using the Prescribed Fire Analysis System. Int J Wildland Fire 19: 1099–1109.

[37] Larsen C (1997) Spatial and temporal variations in boreal forest fire frequency in northern Alberta. J Biogeography 24: 663–673.

[38] Hely C, Bergeron Y, Flannigan M (2000) Effects of stand composition on fire hazard in mixed©\wood Canadian boreal forest. J Veg Sci 11: 813–824.

[39] Yang J, He HS, Shifley SR (2008) spatial controls of occurrence and spread of wildfires in the missouri ozark highlands. Ecol Appl 18: 1212–1225. 18686582 10.1890/07-0825.1

[40] Arienti MC, Cumming SG, Krawchuk MA, Boutin S (2009) Road network density correlated with increased lightning fire incidence in the Canadian western boreal forest. Int J Wildland Fire 18: 970–982.

[41] Liu Z, Chang Y, Chen H, Zhou R, Jing G, et al (2008) Spatial pattern of land surface dead combustible fuel load in Huzhong forest area in Great Xing'an Mountains. Chinese J Applied Ecology 19: 487–493. 18533514

[42] Liu Z, Yang J, Chang Y, Weisberg PJ, He HS (2012) Spatial patterns and drivers of fire occurrence and its future trend under climate change in a boreal forest of Northeast China. Global Change Biol 18: 2041–2056.

[43] Wu Z, He H, Chang Y, Liu Z, Chen H (2011) Development of Customized Fire Behavior Fuel Models for Boreal Forests of Northeastern China. Environ Manage 48: 1148–1157. 21691875 10.1007/s00267-011-9707-3

[44] Bergeron Y, Leduc A, Harvey BD, Gauthier S (2002) Natural fire regime: a guide for sustainable management of the Canadian boreal forest. Silva Fenn 36: 81–95.

[45] Turetsky MR, Kane ES, Harden JW, Ottmar RD, Manies KL, et al (2010) Recent acceleration of biomass burning and carbon losses in Alaskan forests and peatlands. Nature Geosci 4: 27–31.

[46] Rollins MG, Morgan P, Swetnam T (2002) Landscape-scale controls over 20(th) century fire occurrence in two large Rocky Mountain (USA) wilderness areas. Landscape Ecol 17: 539–557.

[47] Johnson E (1996) Fire and vegetation dynamics: studies from the North American boreal forest: Cambridge Univ Pr.

[48] Peterson GD (2002) Contagious disturbance, ecological memory, and the emergence of landscape pattern. Ecosystems 5: 329–338.

