# Supplementary figures and images for: Identifying the Threshold of Dominant Controls on Fire Spread in a Boreal Forest Landscape of Northeast China
Source: PLoS One. 2013 Jan 31;8(1):e55618. doi: 10.1371/journal.pone.0055618 (PMC3561322; doi:10.1371/journal.pone.0055618)

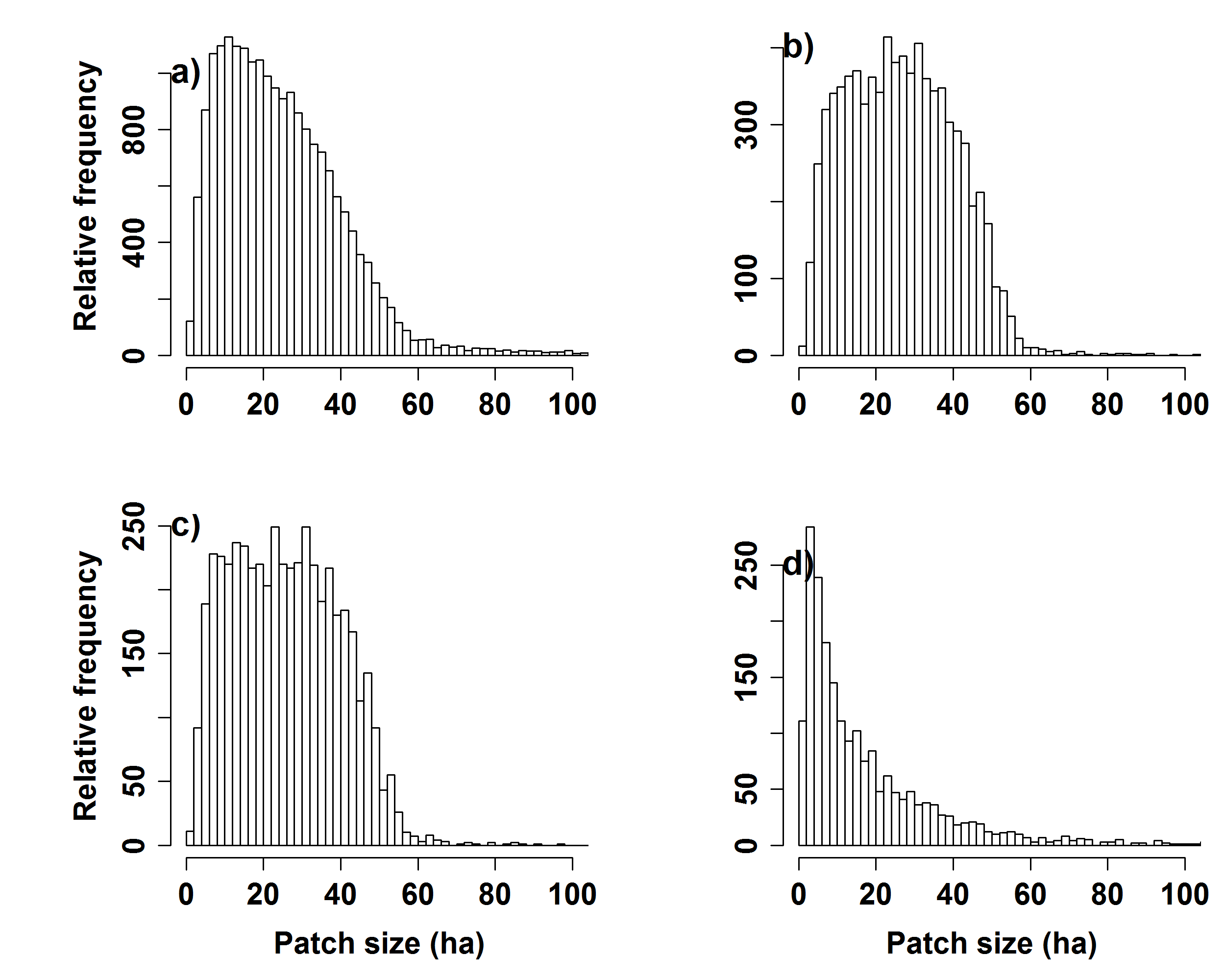

Supplement: Figure S1 — Patch size distribution for a) coniferous; b) mixed; c) broadleaf; and d) meadows within fire scars. (TIF) [file pone.0055618.s001.tif]

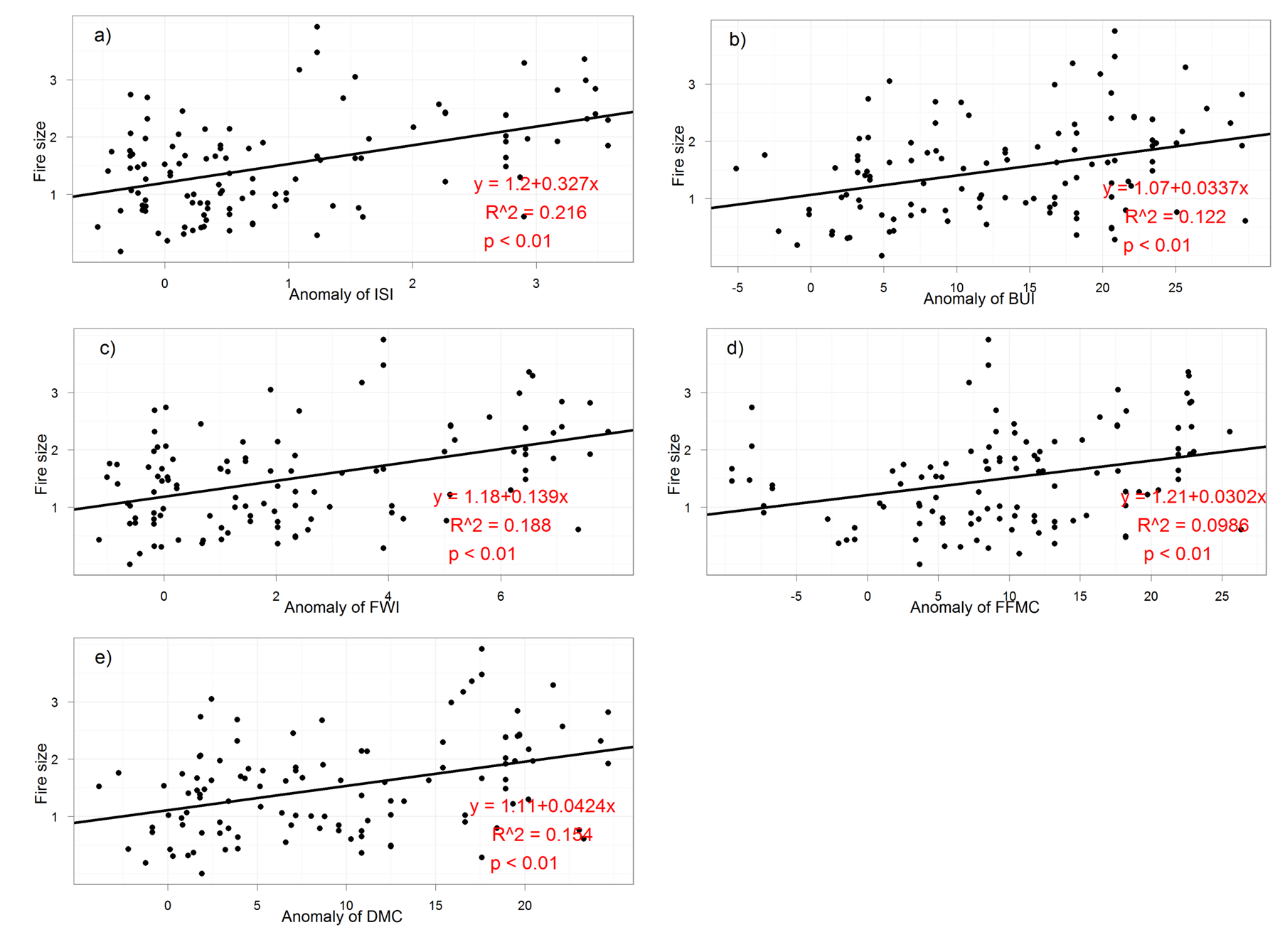

Supplement: Figure S2 — The linear relationship between fire size and anomaly of a) ISI, b)BUI, c)FWI, d)FFMC, and e) DMC. The y axis (fire size) was logarithmic transformed. (TIF) [file pone.0055618.s002.tif]

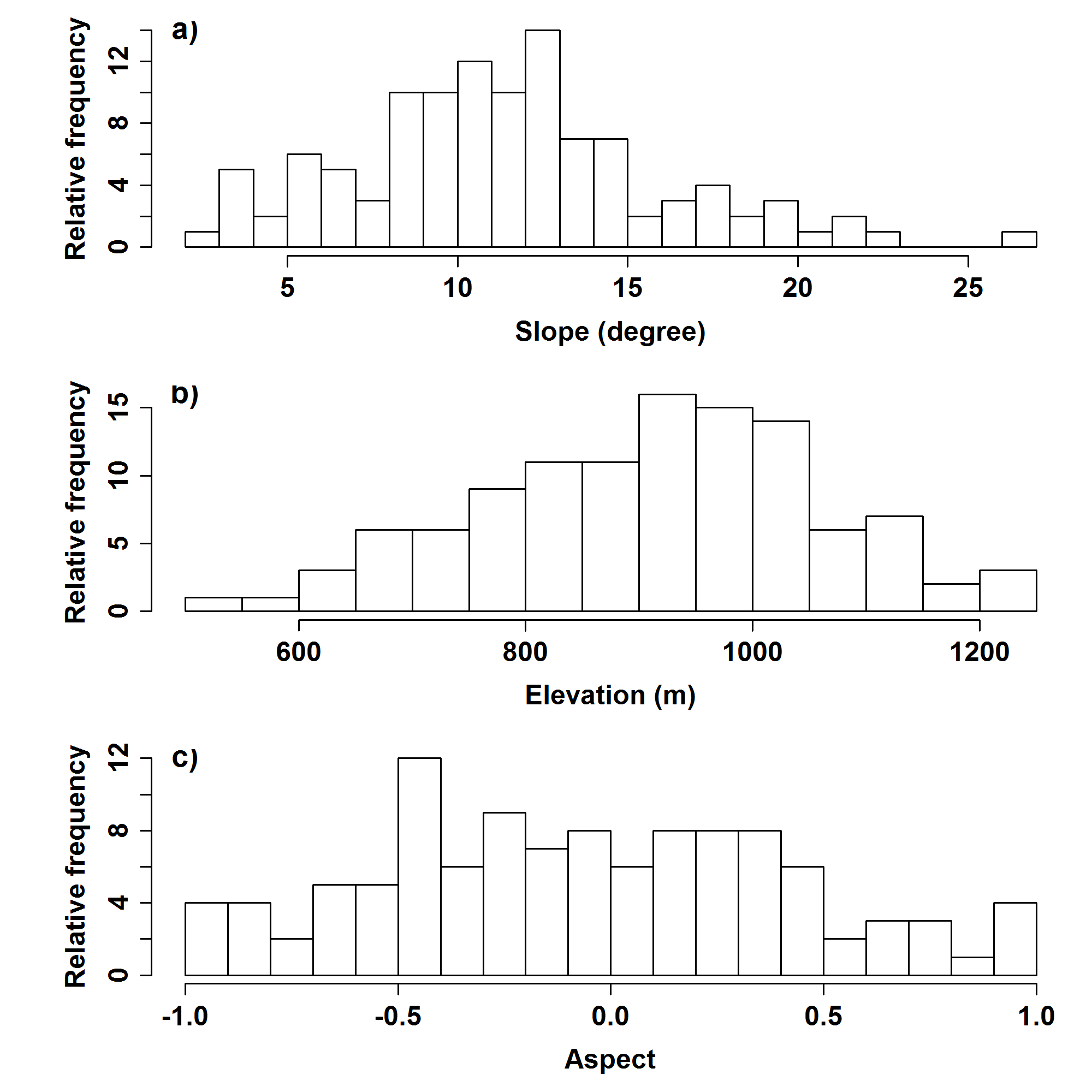

Supplement: Figure S3 — Histogram distribution of a) slope; b) elevation; and c) aspect within fire scars. (TIF) [file pone.0055618.s003.tif]

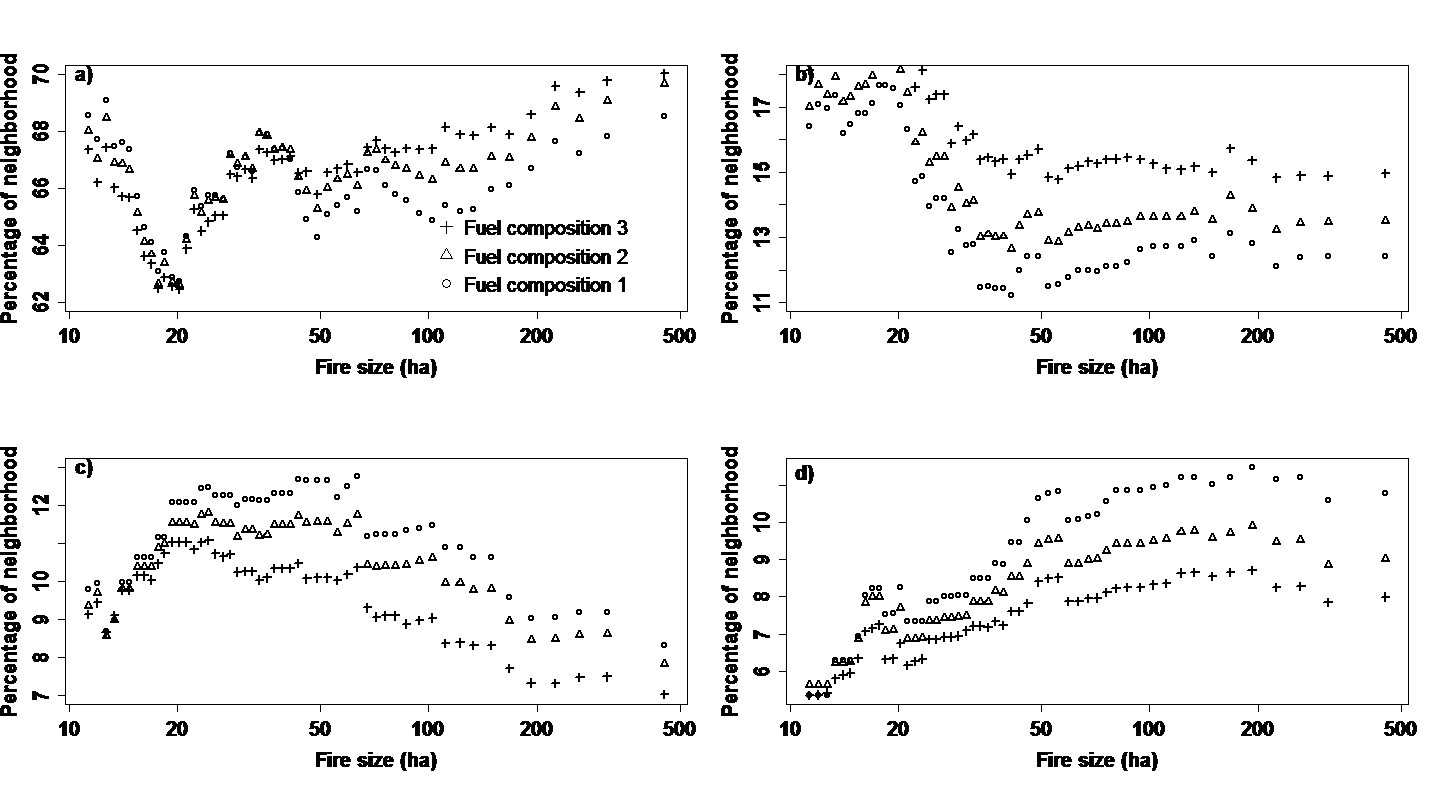

Supplement: Figure S4 — Percentage of a) mixed, b) coniferous, c)broadleaf, and d) meadows within a neighborhood with different size. Fuel composistion 1, 2 and 3 was computed in neighborhood with size of 2, 4, and 8 times of actual fire. The x axis (fire size) was logarithmic transformed. (TIF) [file pone.0055618.s004.tif]

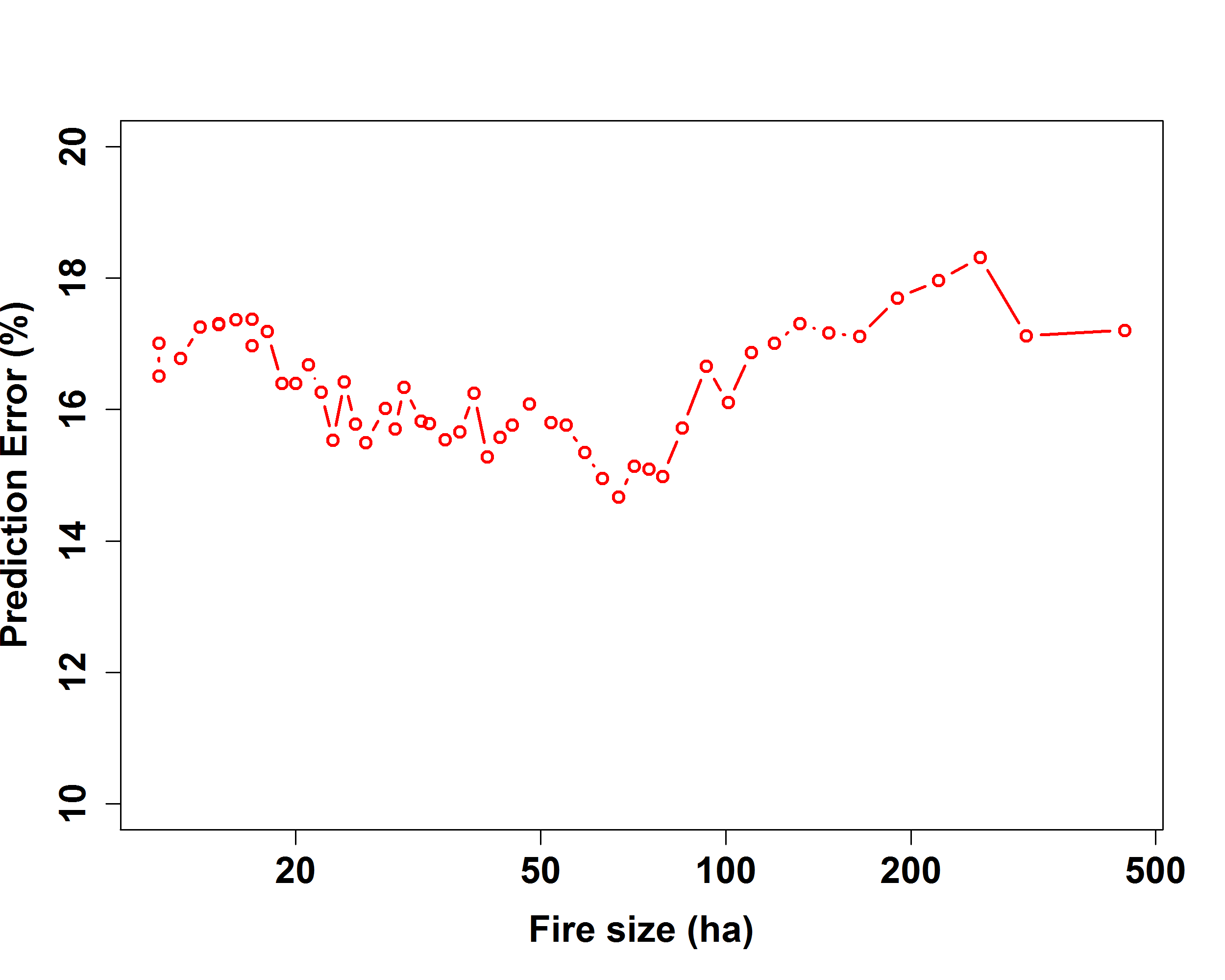

Supplement: Figure S5 — Prediction error of BRT with different fire size. The x axis (fire size) was logarithmic transformed. The prediction error was calculated as follows: Prediction error = (predicted.value - observed.value) ×100/observed.value. (TIF) [file pone.0055618.s005.tif]
